# Methyllycaconitine- and scopolamine-induced cognitive dysfunction: differential reversal effect by cognition-enhancing drugs

**DOI:** 10.1002/prp2.48

**Published:** 2014-06-09

**Authors:** Emile Andriambeloson, Bertrand Huyard, Etienne Poiraud, Stéphanie Wagner

**Affiliations:** Neurofit SAS boulevard Sébastien Brant, Bioparc Parc d’Innovation, 674.00, Illkirch, France

**Keywords:** Alzheimer’s disease, cognitive disorders, donepezil, galantamine, memantine, muscarinic receptors, nicotinic

## Abstract

There is a growing body of evidence pointing to the pivotal role of alpha-7 nicotinic acetylcholine receptor (*α*7 nAchR) dysfunction in cognitive disorders such as Alzheimer’s disease or schizophrenia. This study was undertaken to establish and characterize an in vivo model for cognitive disorder secondary to the blockade of *α*7 nAChR by its specific antagonist, methyllycaconitine (MLA). The results show that MLA elicited cognitive dysfunction as assessed by reduced spontaneous alternation of mice in the T-maze. The maximal effect of MLA produced 25–30% reduction in the spontaneous alternation of mice, a level comparable with that induced by the muscarinic antagonism of scopolamine. Donepezil and galantamine fully reversed both MLA and scopolamine-induced cognitive dysfunction. However, the ED_50_ of donepezil and galantamine was significantly shifted to the left in the MLA- compared to scopolamine-treated mice (0.0005 and 0.002 mg/kg for donepezil; 0.0003 and 0.7 mg/kg for galantamine). Moreover, memantine elicited marked reversion of cognitive dysfunction (up to 70%) in MLA-treated mice while only a weak reversal effect at high dose of memantine (less than 20%) was observed in scopolamine-treated mice. The above findings indicate that MLA-induced cognitive dysfunction in the mouse is highly sensitive and more responsive to the current procognitive drugs than the traditional scopolamine-based assay. Thus, it can be of value for the preclinical screening and profiling of cognition-enhancing drugs.

## Introduction

It is now widely accepted that alpha-7 nicotinic acetylcholine receptor (*α*7 nAChR) plays a central role in cognitive deficits associated with neurodegenerative and cognitive disorders such as Alzheimer’s disease, Parkinson’s disease, and schizophrenia (for review see Conejero-Goldberg et al. 2008; Pohanka 2012). In support of this concept, changes in the brain expression of *α*7 nAChR have been reported in patients with neurodegenerative diseases (Banerjee et al. 2000; Guan et al. 2000; Court et al. 2001; Teaktong et al. 2003; Yu et al. 2005; Counts et al. 2007; Olincy and Freedman 2012). Selective *α*7 nAChR agonists have been reported to improve the cognitive performance of rodents in various assays (Pichat et al. 2007; Roncarati et al. 2009; Sydserff et al. 2009). Moreover, the neuroprotective effect of galantamine and donepezil (approved drugs for the symptomatic treatment of Alzheimer’s disease) has been demonstrated to be mediated by the stimulation of *α*7 nAChR in rat primary neurons (Takada-Takatori et al. 2006a,b; Shen et al. 2010).

The muscarinic antagonism of scopolamine remains the standard method for inducing cognitive deficits in animals and in healthy volunteers despite the accrued data pointing to the importance of *α*7 nAChR in cognitive dysfunction. It thus appears legitimate to develop a preclinical model relevant for pharmacological profiling of new therapeutics targeting the *α*7 nAChR.

Methyllycaconitine (MLA) has been demonstrated as a specific antagonist of the *α*7 nAChR with brain penetrance (Turek et al. 1995; Davies et al. 1999). Numerous studies have implemented MLA as pharmacological tool to demonstrate the specificity of the cognitive response of rodents to *α*7 nAChR agonists (Boess et al. 2007; Pichat et al. 2007; Roncarati et al. 2009; Sydserff et al. 2009). To the best of our knowledge, the potential of MLA to induce cognitive deficit has not yet been studied. Therefore, the present experiments were undertaken to characterize the cognitive dysfunction in the mouse after administration of MLA. Cognitive dysfunction was assessed by the reduced spontaneous alternation of mice in the T-maze. This assay has been previously shown to be sensitive to various pharmacological manipulations affecting memory processes (Gerlai 1998; Spowart-Manning and van der Staay 2004; De Bruin et al. 2010). Furthermore, side by side comparison between cognitive deficit induced by scopolamine and by MLA was also conducted with emphasis to the reversal effect of current cognitive-enhancing drugs, namely donepezil, galantamine, and memantine.

## Materials and Methods

### Animals

Male CD-1 mice, 4–5 weeks old, were obtained from Janvier (Le Genest St Isle, France), and housed 10 per cage in a temperature controlled room (21–22°C) with a reversed light-dark cycle (12h/12h; lights on: 17:30–05:30; lights off: 05:30–17:30) with food and water available ad libitum. They were allowed at least 1 week to acclimatize to the animal facility environment.

### Measure of the cognitive function of mice in the T-maze

T-maze alternation is a widely used behavioral test to assess the cognitive ability of rodents. In this test, the animal alternates between two goal arms during repetitive visit based on the recall of the previously visited arm (Deacon and Rawlins 2006). This alternation capability is reduced by amnesic drugs such as scopolamine (muscarinic receptor antagonist). Several reports have shown that scopolamine-induced decrease in alternation behavior is reversed by various cognitive-enhancing drugs (Bontempi et al. 2003; De Bruin et al. 2010; Maelicke et al. 2010).

The T-maze apparatus is made of gray Plexiglas with a main stem (55 cm long × 10 cm wide × 20 cm high) and two arms (30 cm long × 10 cm wide × 20 cm high) positioned at a 90° angle relative to the left and right of the main stem. A start box (15 cm long × 10 cm wide) is separated from the main stem by a sliding door. Two other sliding doors are present to close off the left or right arm during the forced-choice alternation task. The experimental protocol consists of one single session, which starts with 1 “forced-choice” trial, followed by 14 “free-choice” trials. In the first “forced-choice” trial, the animal is confined 5 sec in the start box and then released while either the left or right goal arm is blocked by the sliding door. After the mouse is released, it will negotiate the maze and eventually enter the open goal arm, and return to the start position. Immediately after the return of the animal to the start position, the closed goal door is opened and the animal is now free to choose between the left and right goal arm (“free-choice trials”). The animal is considered as entered when it places its four paws in the arm. A session is terminated and the animal is removed from the maze as soon as 14 free-choice trials have been performed or 10 min have elapsed, whatever event occurs first.

The apparatus is cleaned between each animal using alcohol (70%). Urine and feces are removed from the maze.

During the trials, animal handling and the visibility of the operator are minimized as much as possible.

The percentage of alternation over the 14 free-choice trials is determined for each mouse and used as an index of memory performance. Spontaneous alternation is defined as entry in a different arm of the T-maze over successive trials (i.e., left–right–left–right, etc.).

### Statistical analysis

Statistical comparisons were made using Statview version 5.0 (SAS Institute, Inc., Cary, NC). To assess the effect of cognitive disruptor agents (scopolamine or MLA), factor effect (treatment) was first tested for significance by ANOVA (Analysis of variance), and then the effect of each dose of disruptor agent was assessed against the level of T-maze performance of vehicle group using Fisher’s protected least significant difference (PLSD) pairwise comparison.

To assess the difference between the two dose-response curves of each cognition-enhancing drug, ANCOVA (Analysis of covariance) was carried out using the treatment with disruptor agents as factor and the doses of cognition-enhancing drug as covariate. A *P*-value level of 0.05 or less was considered significant.

### Drugs

Scopolamine, MLA, and galantamine were purchased from Sigma (Saint-Quentin Fallavier, France). Donepezil and memantine were purchased from Tocris (R&D system, Lille, France).

All drugs were dissolved in sterile 0.9% NaCl and injected to mice at a dosage volume of 10 mL/kg. Scopolamine, MLA, and memantine were administered intraperitoneally (i.p.). Donepezil and galantamine were administered orally (p.o.).

Scopolamine and MLA was administered 40 min prior the start of T-maze trial. Each cognitive-enhancing drug was administered concomitantly with the disruptor agent.

Dose range of each drug was determined in preliminary investigations using doses used in published works (Flood and Cherkin 1986; Dimitrova and Getova-Spassova 2006; Réus et al. 2008; Roncarati et al. 2009).

## Results

### Scopolamine and MLA-induced cognitive deficit in mice

**Figure 1:**
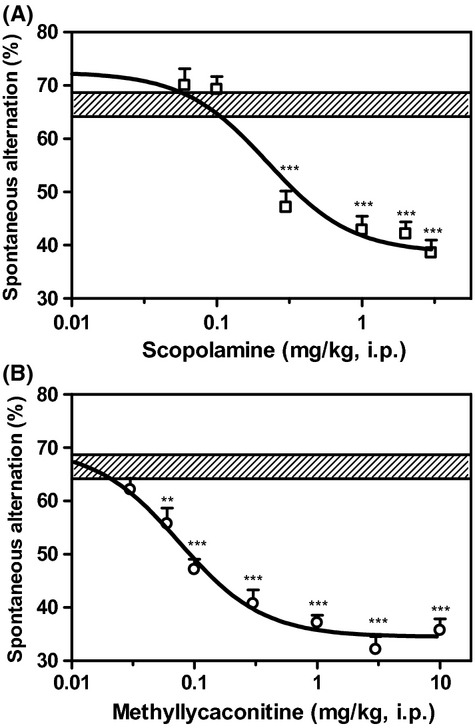
Dose–response curves of scopolamine (A) and MLA (B), showing dose-dependent reduction in spontaneous alternation of mice in the T-maze. Hatched horizontal bar shows the range of performance level of control mice in the T-maze. Data are expressed as mean ± SEM of *n* = 10–12 mice. **P ≤ 0.01; ***P ≤ 0.001, significantly different as compared with the performance of control mice.

As shown in Figure 1, scopolamine and MLA produced a dose-dependent decrease in spontaneous alternation of mice in the T-maze. This suggests that scopolamine and MLA treatment causes cognitive deficit in the mice during the T-maze alternation task. The ID_50_ for each drug was 0.2 and 0.09 mg/kg, respectively. The maximal effect of each drug was comparable (about 30% reduction in the alternation of mice). It is noteworthy that both scopolamine and MLA treatments shorten the time taken to complete the T-maze task (Table 1) in dose-dependent manner, as a result of increased mobility in the T-maze. It was also observed that the alternation of scopolamine or MLA-treated mice was reduced to a degree below 50% chance level. The choice pattern of these mice showed successive insistences on each of two goal arms during the T-maze trial without any significant preference for one particular goal (see Table 2).

**Table 1:** Total time elapsed (min) until completion of the T-maze task

Groups/Doses	Time (min)	SEM	*n*
Vehicle	7.7	0.3	22
Scopolamine (0.06 mg/kg)	6.4**	0.5	10
Scopolamine (0.1 mg/kg)	6.1***	0.3	10
Scopolamine (0.3 mg/kg)	5.3***	0.4	10
Scopolamine (1 mg/kg)	4.8***	0.2	10
Scopolamine (2 mg/kg)	5.3***	0.3	10
Scopolamine (3 mg/kg)	5.3***	0.3	10
MLA (0.03 mg/kg)	7.9	0.4	10
MLA (0.06 mg/kg)	7.7	0.3	10
MLA (0.1 mg/kg)	7.6	0.3	10
MLA (0.3 mg/kg)	6.6*	0.3	10
MLA (1 mg/kg)	6.1***	0.4	10
MLA (3 mg/kg)	5.7***	0.3	10
MLA (10 mg/kg)	5.9***	0.3	10

MLA, methyllycaconitine.

* *P* ≤ 0.05;

** *P* ≤ 0.01;

*** *P* ≤ 0.001, as compared to the Vehicle group.

**Table 2:** Number of entries to each goal arm during the T-maze alternation task

	Entries to right goal arm			Entries to left goal arm		
		
Groups	Mean	SEM	*n*	Mean	SEM	*n*
Vehicle of scopolamine	6.7	0.5	10	7.3	0.5	10
Scopolamine (0.06 mg/kg)	7.2	0.5	10	6.8	0.5	10
Scopolamine (0.1 mg/kg)	6.9	0.4	10	7.1	0.4	10
Scopolamine (0.3 mg/kg)	7.2	1.0	10	6.8	1.0	10
Scopolamine (1 mg/kg)	8.2	0.6	10	5.8	0.6	10
Scopolamine (2 mg/kg)	7.2	0.7	10	6.8	0.7	10
Scopolamine (3 mg/kg)	6.4	0.8	10	7.6	0.8	10
Vehicle of MLA	6.7	0.4	12	7.3	0.4	12
MLA (0.03 mg/kg)	6.6	0.4	10	7.4	0.4	10
MLA (0.06 mg/kg)	6.5	0.4	10	7.5	0.4	10
MLA (0.1 mg/kg)	6.6	0.4	10	7.4	0.4	10
MLA (0.3 mg/kg)	6.7	0.6	10	7.3	0.6	10
MLA (1 mg/kg)	6.6	0.5	10	7.4	0.5	10
MLA (3 mg/kg)	6.4	0.5	10	7.6	0.5	10
MLA (10 mg/kg)	6.8	0.3	10	7.2	0.3	10

MLA, methyllycaconitine.

In the remaining experiments where the reversal effect of cognitive-enhancing drugs (donepezil, galantamine, and memantine) was tested, scopolamine and MLA were used at doses that produce about 80% of their maximal effect (1 and 0.3 mg/kg, respectively).

### Reversal of cognitive deficit by donepezil

**Figure 2:**
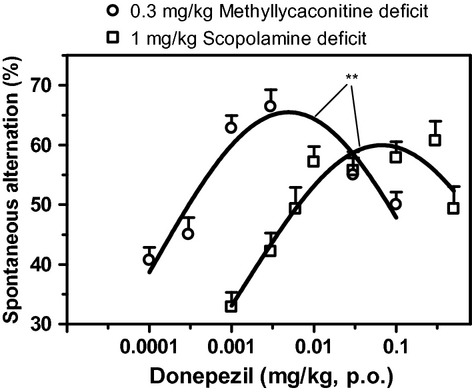
Dose–response curves of donepezil reversal of a scopolamine-induced deficit (square symbol) and an MLA-induced deficit (circle symbol) as assessed by the change in spontaneous alternation of mice in the T-maze. Data are expressed as mean ± SEM of *n* = 10 mice. **P ≤ 0.01, indicate significant difference between curves.

Figure 2 shows that donepezil produced a dose-dependent increase in spontaneous alternation of both scopolamine- and MLA-treated mice, suggesting a reversion of the cognitive deficit. The maximal increase in spontaneous alternation induced by donepezil did not statistically differ between scopolamine- and MLA-induced cognitive deficit (27 and 22% increase, respectively). However, in the scopolamine-induced cognitive deficit, the ED_50_ of donepezil was four times greater than in the MLA-induced deficit (0.002 and 0.0005 mg/kg, respectively). Furthermore, the reversal effect of donepezil in the scopolamine-induced deficit showed a decline at the dose of 0.5 mg/kg. This decline was also observed in MLA-induced deficit but only occurred at doses 15 times lower than in scopolamine-induced deficit. It is noteworthy that up to the dose of 0.5 mg/kg, the treatment with donepezil did not induce any significant change to the time taken by scopolamine-treated mice to complete the T-maze alternation task (data not shown).

### Reversal of cognitive deficit by galantamine

**Figure 3:**
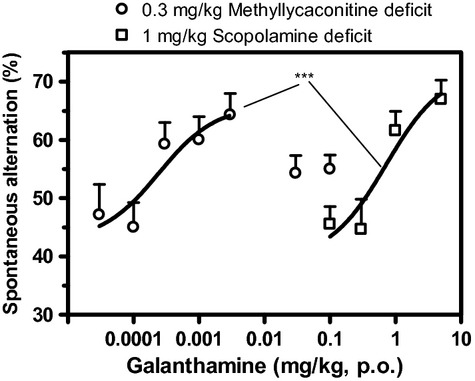
Dose–response curves of galantamine reversal of a scopolamine-induced deficit (square symbol) and an MLA-induced deficit (circle symbol) as assessed by the change in spontaneous alternation of mice in the T-maze. Data are expressed as mean ± SEM of *n* = 10 mice. ***P ≤ 0.001, indicate significant difference between curves.

Galantamine elicited a dose-dependent increase in the spontaneous alternation of both scopolamine- and MLA-treated mice (Fig. 3). The maximal increase in spontaneous alternation induced by galantamine did not statistically differ between scopolamine- and MLA- induced cognitive deficit (21% and 25% increase, respectively). In the scopolamine-induced cognitive deficit, the ED_50_ of galantamine was 0.7 mg/kg. However, the ED_50_ in the MLA-induced deficit was reduced by 3.5 orders of magnitude (0.0003 mg/kg) as compared to that in scopolamine-induced deficit. Furthermore, the reversal effect of galantamine in the scopolamine-induced deficit did not show any decline up to the dose of 5 mg/kg. In contrast, decline was observed in MLA-induced deficit at the dose of 0.03 and 0.1 mg/kg. It is noteworthy that up to the dose of 5 mg/kg, the treatment with galantamine did not induce any significant change to the time taken by scopolamine mice to complete the T-maze alternation task (data not shown).

### Reversal of cognitive deficit by memantine

**Figure 4:**
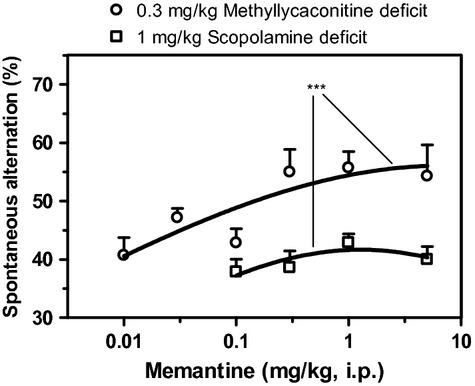
Dose–response curves of memantine reversal of a scopolamine-induced deficit (square symbol) and an MLA-induced deficit (circle symbol) as assessed by the change in spontaneous alternation of mice in the T-maze. Data are expressed as mean ± SEM of *n* = 10 mice. ***P ≤ 0.001, indicate significant difference between curves.

As shown in Figure 4, memantine did not produce any significant increase in spontaneous alternation of scopolamine-treated mice. In contrast, memantine elicited up to 18% increase in spontaneous alternation of MLA-treated mice. The ED_50_ of memantine in reversing MLA-induced cognitive deficit was 0.09 mg/kg. Up to the dose of 5 mg/kg, memantine did not significantly modify the time taken by scopolamine mice to complete the T-maze alternation task (data not shown).

## Discussion

In this study, we sought to characterize the cognitive dysfunction induced by MLA, a specific antagonist of the *α*7 nAChR. Cognitive function was assessed by measuring spontaneous alternation of mice in the T-maze, a test known to respond to various pharmacological manipulations. We found that MLA elicited marked reduction in the spontaneous alternation of mice in the T-maze which suggests a cognitive deficit. The maximal effect of MLA produced 25–30% reduction in the spontaneous alternation of mice, a level comparable with that induced by the muscarinic antagonism of scopolamine in the same paradigm. Furthermore, similarly to scopolamine, MLA treatment was associated with a hypermobility of mice in the T-maze. These results indicate that MLA produced cognitive dysfunction in the mouse comparable to that of the standard drug scopolamine.

Noteworthy that the alternation of scopolamine or MLA-treated mice was reduced to a degree below 50% chance level as a consequence of successive insistences on each of two goal arms. The same phenomenon has been reported by Gerlai (1998) in mouse T-maze task following hippocampal lesion and the author concluded that the choice of mice with hippocampal dysfunction does not operate in alternating fashion but rather in a repetitive, stereotypical way. Based on the similarity of behavior in the T-maze alternation, it is very plausible that treatment with scopolamine and MLA in this study alters the hippocampal function of mice. Interestingly, stereotypic and repetitive behaviors are symptoms observed in frontotemporal dementia and to a lesser extent in Alzheimer’s disease (Nyatsanza et al. 2003).

MLA has been shown to cross the blood brain barrier and penetrates to the brain following systemic administration in rat (Turek et al. 1995; Lockman et al. 2005). In addition, Davies et al. (1999) reported high binding site density of [^3^H]MLA in hippocampus and hypothalamus of rat brain. The T-maze continuous alternation task has been shown to rely on the hippocampal function in mouse and rat (Gerlai 1998). In this study, the cognitive deficit induced by MLA in the T-maze paradigm is most probably associated with an alteration of the hippocampal function of mice.

To the best of our knowledge, this is the first report of MLA-induced cognitive deficit in the mouse T-maze alternation task. This deficit is most plausibly mediated by inhibition of *α*7 nAChRs. Indeed, MLA has been described as a potent competitive antagonist, selective for *α*7 nAChRs in the nanomolar range (Ward et al. 1990; Wonnacott et al. 1993). In addition, the involvement of muscarinic receptors in MLA-mediated cognitive deficit can be ruled out since MLA does not show any affinity for muscarinic receptors up to 0.1 mmol/L in rat brain (Ward et al. 1990). Furthermore, various pharmacological block studies have demonstrated that MLA suppresses the memory-enhancing effect of selective *α*7 nAChR agonists in social recognition and object recognition tasks in the rat (Boess et al. 2007; Pichat et al. 2007). These come in line with our finding where the MLA-induced cognitive deficit was fully blocked by PNU-282987, a selective *α*7 nAChR agonist (Hajós et al. 2005) (data not shown). Altogether, these findings suggest that the *α*7 nAChR pathway is involved in the cognition process in rats or mice.

In this study, the potential clinical relevance of MLA-induced cognitive deficit was supported by the fact that three procognitive drugs approved for the clinical treatment of cognitive disorders, namely donepezil (acetylcholinesterase [AChE] inhibitor), galantamine (AChE inhibitor and allosteric modulator of nAChR) and memantine (antagonist of N-Methyl-D-aspartate [NMDA] receptors), reversed the symptoms of cognitive impairment in mice. In parallel, the comparison with the traditional scopolamine-based assay was undertaken. The results showed that while donepezil and galantamine reversed both MLA- and scopolamine-induced cognitive deficit, their ED_50_ were dramatically reduced (by 1 and 3 orders of magnitude, respectively) in the MLA- compared to the scopolamine-based assay. Moreover, memantine elicited marked reversion of cognitive deficit in MLA-based assay whereas it was nearly ineffective in the scopolamine-based assay. These results suggest that MLA-induced cognitive deficit positively responds to the current cognition-enhancing drugs and it shows higher sensitivity than the model of scopolamine-induced deficit.

Sensitivity of a model is critical in translating animal data to first-time-in-human dose selection. It is logical to estimate the human dose from the most sensitive system available. Since to date scopolamine-induced deficit is the most used preclinical model (to some extent also clinical) for the profiling of procognitive drugs, it is thus tempting to speculate that the dose used for these procognitive drugs could be overestimated. As shown, the results of this study indicated that the optimal dose of donepezil (0.1 mg/kg) in the scopolamine-deficit model showed a marked decline of efficiency in the MLA-deficit model. It is well known that the higher the dose of a drug, the greater the risk for potential side effects that could compromise or alter its efficiency.

Donepezil and to some extent galantamine are known to increase the short lifespan of acetylcholine (ACh) through inhibition of AChE, the enzyme responsible of ACh breakdown. AChE inhibitors lead to an increased brain level of ACh in mice and rats (Naik et al. 2009). ACh is secreted within the synapse and it acts as an agonist of both muscarinic and nicotinic receptors. However, the affinity for muscarinic receptors (Gurwitz et al. 1985; Kellar et al. 1985) is much greater than the affinity for nicotinic receptors (Marks et al. 1986; Anand et al. 1993; Gopalakrishnan et al. 1995; Jensen et al. 2003) (nanomolar range and micromolar range, respectively). Therefore, unless muscarinic receptors are blocked, muscarinic rather than nicotinic responses predominate.

It is thus hypothesized that the difference in potency of donepezil or galantamine toward scopolamine- and MLA- deficit accounts for the different affinity of ACh toward muscarinic and nicotinic receptors, whichever is available. In the presence of MLA which impairs the *α*7 nAChR pathway, lower level of ACh (hence lower dose of donepezil or galantamine) is sufficient to produce greater cognitive effect via stimulation muscarinic acetylcholine receptor (mAChR) pathways. In contrast, when mAChRs are impaired in the presence of scopolamine, a level of ACh greater than the above case (hence higher dose of donepezil or galantamine) are required to obtain comparable cognitive effect via stimulation of nAChR pathway. Schematic illustration of the proposed mechanistic model is provided as supplementary data.

It is noteworthy that the difference in the potency of galantamine in reversing scopolamine- versus MLA-induced deficit was much greater (about three times) than that observed for donepezil. This can be explained by the reported dual effect of galantamine. Indeed, galantamine has been reported to act as nicotinic allosteric potentiating ligand in addition to its AChE inhibitor effect (Samochocki et al. 2003). This allosteric potentiation of nicotinic receptor has been shown to occur at low but not high concentration of agonist, that is, endogenous ACh (Schrattenholz et al. 1996; Santos et al. 2002; Samochocki et al. 2003) as well as at low concentration of galantamine (Schrattenholz et al. 1996; Samochocki et al. 2003). Thus, the allosteric effect of galantamine on nAChRs enhanced nicotinic responses with the same ACh increase compared to donepezil and result in a greater ED_50_ shift in scopolamine- versus MLA-based assay.

The cognitive-enhancing effect of memantine is not very clear yet and results reported so far are not always consistent. Indeed, some authors reported that memantine improves memory in rats or mice and others demonstrated that it lacks cognitive effect or only shows minor effect. Memantine is believed to mediate its cognitive action through a nonspecific antagonism of NMDA receptor which results in a restoration of homeostasis in the glutamatergic system necessary for cognition processes. Memantine has also been found to block other receptors such as nicotinic receptor, 5-HT-3 receptors (for review see Parsons et al. 2007). However, the blockade of the all above mentioned receptors could not explain why memantine was effective in reversing MLA- but not in scopolamine- induced cognitive deficit. Drever et al. (2007) reported actions of memantine beyond NMDA receptor antagonism, including stimulating effects on cholinergic signaling via muscarinic receptors. In this study, such a stimulation of muscarinic receptors by memantine is possible in the presence of MLA but most probably not when the muscarinic antagonism of scopolamine is present. Indeed, Drever and coworkers have shown that memantine enhances the synaptic transmission in hippocampal brain slice, an effect blocked by scopolamine. Therefore, stimulation of the muscarinic receptor appears as the most adequate hypothesis to explain the differential response of memantine with respect to the reversion of MLA- and scopolamine- induced cognitive deficit.

Altogether, the data of this study highlight the involvement of *α*7 nAChR signaling in the cognition process. It also supports previous studies which suggest the critical role of *α*7 nAChR dysfunction in the cognitive disorders. Finally, the animal model that mimics *α*7 nAChR dysfunction implemented herein could have potential clinical relevance distinct from that of the traditional model of muscarinic receptors dysfunction.

## Abbreviations

ACh: acetylcholine
AChE: acetylcholinesterase
ANOVA: analysis of variance
*α*7 nAChR: alpha-7 nicotinic acetylcholine receptor
MLA: methyllycaconitine

## Supporting Information

**Data S1.** Proposed model of the mechanism.
