# Supplementary material for: Methyllycaconitine- and scopolamine-induced cognitive dysfunction: differential reversal effect by cognition-enhancing drugs
Source: Pharmacol Res Perspect. 2014 Jun 9;2(4):e00048. doi: 10.1002/prp2.48 (PMC4186438; doi:10.1002/prp2.48)
Supplement: Supplementary file 1 — Data S1. Proposed model of the mechanism. [file prp20002-e00048-SD1.doc]

Supplementary data


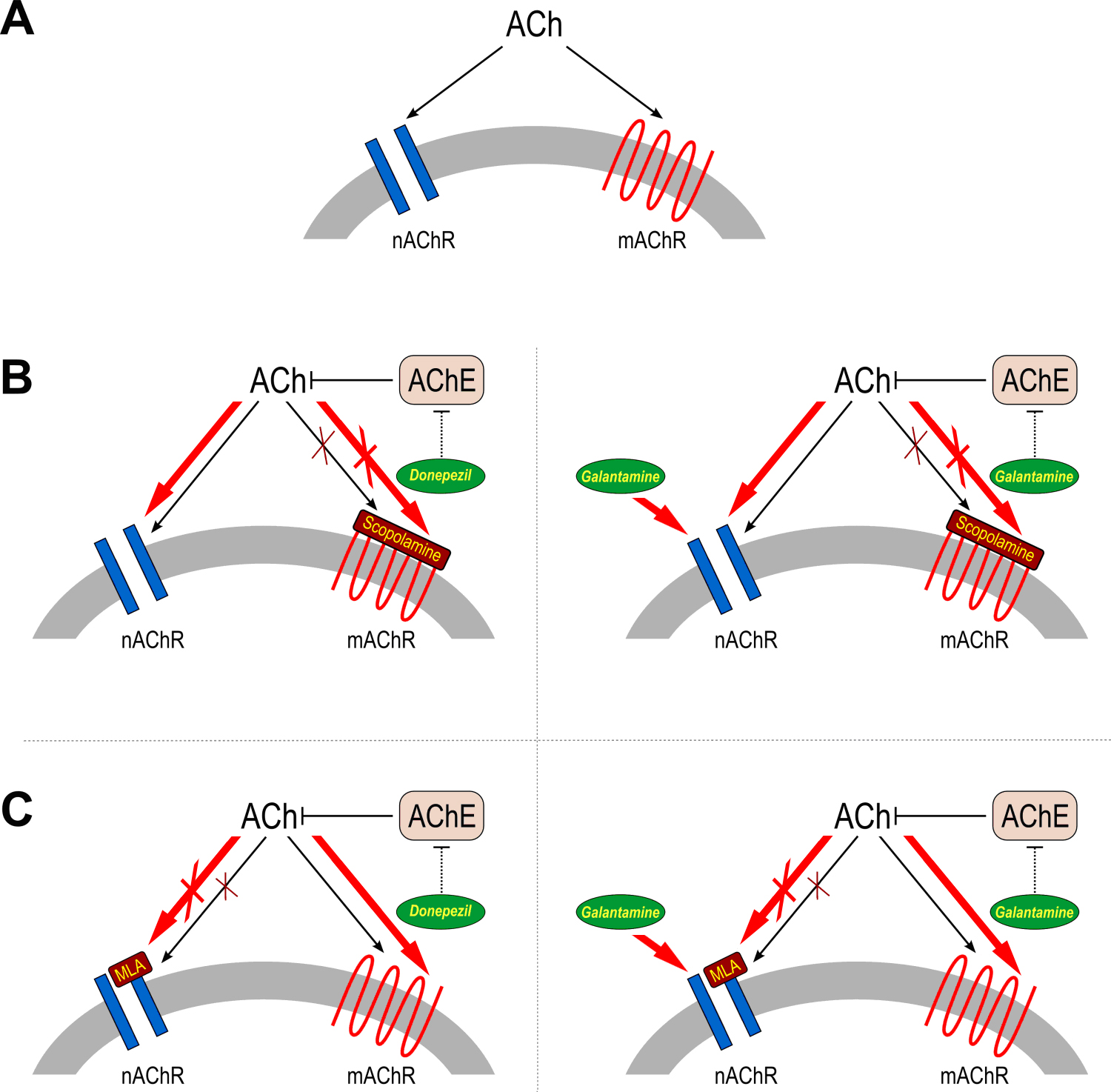


Proposed model of the mechanism of reversal of MLA- and Scopolamine-induced cognitive impairment by Donepezil and Galantamine. Cognition-impairing inhibitors are in red rectangles, procognitive drugs are in green ellipses. Black arrows indicate stimulation of receptors by ACh in the absence of any pharmacological manipulations, red bold arrows are direct or indirect effects of pro-cognitive drugs, red crosses show effects of scopolamine or MLA.

***A***, In the absence of pharmacological manipulations, ACh activates both nAChRs (ligand-gated channels) and mAChRs (G-protein coupled receptors). Pro-cognitive cholinergic effects are well known to be mediated by both nicotinic and muscarinic ACh receptors as shown by earlier studies and exemplified by the experiment in figure 1 of the present study (Inhibition of either nAChRs or mAChRs results in cognitive impairment, Fig.1).

***B***, Mechanism of action for Donepezil (*left*) and Galantamine (*right*) in scopolamine-induced cognitive impairment. Disruption of mAChR-mediated signalling by scopolamine induces cognitive impairment. Donepezil inhibits AChE which leads to increase in ambient ACh concentrations and prolongation of ACh stimuli due to delayed ACh removal. Increased ACh concentrations produce greater nAChR-mediated responses and prolonged ACh exposures may recruit more remote nAChRs (red bold arrow between ACh and nAChR) which leads to compensation of scopolamine-induced cognitive impairment. The inhibitory effect of galantamine on AChE produces the same effect which is aided by its direct positive allosteric modulation of nAChRs (red bold arrow between galantamine and nAChR). Galantamine’s positive allosteric activity augments nAChR-mediated currents contributing to the reversal of cognitive deficit (red bold arrow between galantamine and nAChR). ***C***, Mechanism of action for Donepezil (*left*) and Galantamine (*right*) in MLA-induced cognitive impairment. Disruption of nAChR-mediated signalling by MLA (mainly α7 nAChR) induces cognitive impairment. Donepezil inhibits AChE which leads to increase in ambient ACh concentrations and prolongation of ACh stimuli due to delayed ACh removal. Firstly, elevated ACh increases signalling through mAChRs which otherwise would be blocked in the scopolamine model. Secondly, despite the blockade of nAChR by MLA, increased ACh concentration produces greater responses in the non-blocked population of nAChRs since MLA is a competitive antagonist, to some extent relieves MLA blockade. Note that the difference in the affinity of ACh towards nAChR and mAChR most plausibly accounts for increased sensitivity (lower ED_50_, See Figures 2-3) of procognitive compounds in the MLA-based test. In addition, the synergistic action nAChR and mAChR pathways should, theoretically, produce a greater reversal of MLA-induced cognitive impairment.

Galantamine produces the same effects on nAChRs and mAChRs due to inhibition of AChE and increase in ACh concentration. However, its additional effect is potentiation of nAChRs by positive allosteric modulation. Thus the additional, allosteric, effect of galantamine on nAChRs may produce a better reestablishment of nicotinic transmission with the same ACh increase compared to Donepezil and result in a greater ED_50_ shift in scopolamine- vs. MLA-based test (See Figure 3)
